# Discussion on the "Retention of Artificial Dentures in Edentulous Lower Jaws."

**Published:** 1894-08

**Authors:** 


ARTICLE VI.

### DISCUSSION ON THE "RETENTION OF ARTIFICIAL DENTURES IN EDENTULOUS LOWER JAWS."

Mr. David Hepburn opened the discussion. Having remarked upon the advantage of occasionally displacing a paper by a discussion on some practical subject, he stated that in introducing the subject for which he made himself responsible, he intended to confine himself to a consideration of those cases which present themselves happily only from time to time, which gives much uneasiness to the patient and specially tax the ingenuity of the practitioner, cases which for want of a more elegant term he would call "slipping lowers." The difficulty then upon which they wished to throw light was that which revealed itself when a patient, probably advanced in years, seeks the aid of the dental practitioner with a view to being supplied with artificial teeth, all the natural ones being lost and not even the roots remaining. A denture is adapted, but as the early days of trial progress it is found that it has a tendency to slip forward, in consequence of which the mucous membrane becomes irritated, and more or less ulceration and pain is set up. These symptoms are relieved by "easing," as it is called, but only temporary relief results; shortly fresh spots of ulceration appear, and the operation of "easing" has to be repeated. What were the conditions leading to such a result? It would be well to recall some of the simple anatomical facts connected with the lower jaw which had a direct bearing on the subject. The inferior maxillary bone varies much at different periods of life. It consists of a curved horizontal portion and two perpendicular portions (the rami), which join the posterior portion of the body on either side. At birth the rami join the body at an oblique angle, as maturity advances, and up to middle life, the obliquity diminishes until the junction is rectangular, in extreme old age, or second childhood, obliquity is resumed, simulating the condition as presented at birth. It was this obliquity in a more exaggerated form which rendered the jaw intolerant of the artificial denture. Comparing the normal outline of the jaw in middle life with that of typical old age, he would draw attention to the position of the mental foramen, noticing it first from its external aspect. It would be observed to be midway between the superior surface of the alveolus and the base of the jaw in the first case, in the second, the alveolus having disappeared, it opened close to the superior surface of the body of the jaw, at which point its nerves and vessels spread out directly to the mucous membrane. Viewing laterally the internal surface of the inferior maxilla, the mylohyoid ridge would be seen to be directly midway between the superior surface of the alveolus and the base of the jaw. Again, a further point to be noticed on this aspect of the jaw was the group of genial tubercles situated on the inner side of the symphisis, when extreme absorption had taken place they often assumed an indefinite mass and form with their tendinous attachments, on which no denture can rest. An inclined plane is created laterally, and from this eminence a denture will often slip downward and forward. In addition to these immediate aspects of the body of the jaw itself there were conditions of mobility different from, or rather an exaggeration of, those which the joint is capable of performing during the period when the teeth are existent. As most bony prominences become modified with advancing years so it is with this one. Further, all these senile changes were frequently associated with alterations in the spinal column, resulting in a fixed downward and forward position being imparted to the head, causing the jaw to rest most naturally in a state of protrusion. By reason of this space posteriorly is reduced to a minimum, therefore when the artificial teeth are present there is a tendency for the denture to be tilted and displaced. Affecting also lower dentures, they had the muscular force of the tongue. The points he had endeavoured to emphasize might exist temporarily or permanently. With regard to treatment, provided that careful impressions of the part had been obtained, and the most natural antagonism ascertained, the question presented itself as to whether or not spiral springs should be employed. He would suggest that they should be dispensed with, as, by reason of the configuration of the lower jaw, they will tend to displace, rather than retain, the denture. At the same time he thought there existed certain classes of cases which needed the employment of springs where the retention of the denture by suction was impossible. When necessity arises for the fitting of springs certain points must be adhered to: in view of the limited space they must be brought well forward; further, it should be provided that when the jaw opens the lower swivel will be on a vertical line with the upper swivel, in this position they should be "blocked" *i.e.*, stops should be inserted in proximity to the swivel heads. By this means the direct thrust of the spring will be maintained. Deeply cut chambers will sometimes suffice to procure the same result. The consideration of the adjustment of springs would afford a theme in itself for discussion. Dental surgeons will receive eagerly any suggestion which would retain lower dentures in their place, and by the kindness of Mr. Lennox, of Cambridge, he (Mr. Hepburn) was able to show the contrivance which was fully described by Mr. Lennox at the exeter meeting. The most heroic treatment was that suggested by Mr. Dall, of Glasgow, who created sockets in the lower jaw by drilling, into which he inserted posts, by means of which the denture is retained. Another method was that of weighting lower dentures to ensure stability, the principle, Mr. Hepburn thought, of the greatest service where the muscles were strong, but he had a case where the muscular resistance was so great that the denture was like "a storm tossed vessel in an angry sea." With regard to the relief of pain by lining the denture with gutta-percha, the mucous membrane became absolutely tolerant of it, but there was the disadvantage of impurity after prolonged use. The metal moulds required some ingenuity in their treatment. The perishable nature of the base, and the impossibility of paring it away without destroying its surface should laceration occur, detracted from its advantages, and all things considered, vulcanite seemed the best, In conclusion, he would give his own experience in a few words. He believed that the difficulties could only be overcome by personal adjustment of the denture to the mouth itself through repeated trials, consequently, during the initial stages of "fitting," frequent visits at short intervals on the part of the patient were of the utmost importance. Having examined the patient, and formed a prognosis of the difficulties to be dealt with, impressions should be taken, preferably in plaster of Paris. When these were cast, and trial plates prepared, the approximate articulation should be taken, with the head bent slightly forward. The bite having been taken, the upper denture might be made and the lower one set in wax, keeping the lower front teeth well within the upper teeth as to position and height; if springs were used they should be temporarily adjusted. Almost invariably the articulation, notwithstanding the precautions, would be found at fault—the lower would slip forward. He would then cover the crowns of the teeth with a moulding of pink wax while the wax was soft; taking the dentures out of the mouth, the two should then be united according to the indications thus obtained; the bite might then be finally adjusted. In doing this the teeth should again be set well back, and no prominent cusps be allowed to interfere with the free movement of the jaws. In the upper denture additional canines in the place of first bicuspids would allow of freer movement without tilting.

Mr. Lawrence Read said, with regard to the remarks of Mr Hepburn as to the lining of the lower pieces, this was a matter in which he had taken some interest, and had devoted a good deal of time to some six or seven years ago; the difficulty of finishing the rubber he had now quite overcome, it was a very simple matter when once one knew how to deal with it. His method was to take a large peice of steel wire, about a quarter of an inch thick with a rounded end, make it red hot and run it over any rough surface that it was desired to polish. If it was then treated with a little chloroform on wool a beautiful polished surface was left, just as if it had been baked in a metal mould. It took no time to do, and every edge could be finished off as nicely as could be desired. He had had somd lowers with the soft rubber linings, which had been worn for seven years, and were still perfect in every way. It was not at all necessary to make them on metal moulds, and they could be finished off, if a little rough, just as readily as a piece of hard vulcanite could be polished.

Mr. S. A. Coxan (Wisbech) demonstrated, by means of diagrams on the black-board, the plan he adopted in order to get the swivels for springs exactly opposite one another. His method was to take a small strip of No. 7 gold, fold another piece over it, pressing it tightly, thus forming a bolt which could be drawn either forward or backward, and permitted of the springs being put in absolutely true. Another advantage was that the swivel could be attaced without getting twisted.

Mr. F. J. Bennett. commenting on the model shown and explained by Mr. Hepburn as being in an oblique position an inclined plane being formed, thought that the condition would bear another interpretion. As it stood, undoubtedly there was an inclined plane, but he (Mr. Bennett) doubted whether in nature they ever got that condition of the mouth if left to itself. If artificial teeth were placed in the mouth it might be so, but without them there was no longer an inclined plane, the lower and upper jaws became parallel, He thought that these facts should teach them to make the bite as shallow as possible, in that way the inclined plane would be reduced to a minimum, Mr. Bennett then criticised at some length the anatomical accuracy of the diagrams by which Mr. Hepburn's opening was illustrated.

Mr. G. Brunton said there was a point which he had been expecting to hear mentioned, namely, that where springs were worn they not unfrequently twisted round, in consequence of which the upper denture is twisted in one direction and the lower in another. He might say that he personally very seldom used spiral springs, but he had seen such cases, and it occurred to him that the reason of the twisting was that the two spiral springs were both coiled in the same direction; he thought that if they were coiled in opposite directions the difficulty would be overcome.

The President remarked that he had himself seen cases similar to those alluded to by Mr. Brunton. They were usually cases where the denture had been worn for many years, and he was inclined to attribute the twisting to the absorption of the alveolus at the sides leaving the plate nearly impinging on the palate in the middle. He had never seen the twisting in freshly made plates.

Mr. W. A. Vice narrated an instance which had come under his notice only the previous week, and which supported the view just expressed by the President. The denture had been worn for a long time, the lower arch was absorbed very much until the lower piece would not fit at all. The lady dated the movement round to the left side from the time of having a new spring on the right, and Mr. Vice thought that she was probably correct; there certainly was some difference in the strength of the springs, the old one on the left being much weaker than the new one on the opposite side.

Mr. S. J. Hutchinson said that Mr. Hepburn had spoken of the loose membrane which was sometimes the cause of trouble in fixing a lower denture. His usual plan was to snip off the membrane with a pair of scissors, allowing the wood to cicatrize, and in this way the difficulty was overcome most satisfactorily.

Mr. H. Baldwin narrated particulars of a case in his own practice, presenting some very interesting features. It was an instance in which an artificial denture, fitted to an edentulous mouth, hurt the patient, not because it slipped forward but because it moved about so much. But little remained of the lower alveolar process. The attachments of genio-hyoglossus and mylohyoid muscles stood up from the general level of the lower jaw. It was an interesting point, Mr. Baldwin thought, that these were preserved when there had been the greatest possible removal of the rest of alveolar process. To make the plate more comfortable velum rubber was tried as lining, but it had to be given up as the patient, a man aged sixty, found it induced a tendency to champ his jaws together. The greatest success in treating this case was ultimately obtained by setting up the teeth on a Bonwill articulator, paying great attention to the practical hints upon which Dr. Bonwill laid stress, that was to say taking care to have the line of articulation of the two rows of back teeth bending decidedly upwards, and further providing that in all possible movements of the lower jaw the lower denture would strike the upper one at *three points* at once, Considerable care and trouble was necessary in order that this requirement should be fulfilled, but it could not be done.

Mr. W. A. Maggs thought that perhaps Mr. Hepburn laid too much stress upon the mandible, personally he had always regarded the articulation as responsible for the forward movement. The fact that the *eminentia articularis* in aged skulls was so much diminished would tend of course to the free movement of the mandible. No doubt the articulation became very lax, and if it were possible to examine a sufficient number of skulls a considerable difference in the neck of the condyle, and probably in the condyle itself, would be found. He thought that the cases just metioned by Mr. Baldwin were probably aggravated by the continuous wearing of the denture.. While it was difficult to do without the teeth, yet they knew that continuous pressure produced absorption, and he thought that if the denture were not so constantly worn the tendency to wasting of the jaw would be diminished.

Mr. J. H. Badcock said that in cases where one had great difficulty in making a lower remain in its place, a model taken in the ordinary gutta-percha or stent would show a ridge all round, if, however, a model of the same jaw were taken in plaster of Paris they would find no ridge; for this reason he strongly advocated plaster of Paris in preference to gutta-percha for taking impressions in such cases. If the model were taken so as to rest only on the floor of the mouth, and not at all upon the bulging sides, there would be very little difficulty, and there would also be much less trouble *afterwards* in easing the case away where necessary. With regard to smoothing the velum rubber with a hot iron, he thought the whole secret of success lay in haviug the iron hot enough.

Mr. R. H. Woodhouse did not think that the discussion ought to pass without an allusion to the extreme importance of preserving the natural teeth. They knew that the alveolus was so subservient to the presence of the natural teeth that the retention of even a single tooth, if prolonged, might save all the trouble that had been described that evening.

Mr. W. Hern desired to touch upon a point with regard to the muscular attachments referred to in the mylohyoid ridge. He thought it would be found a considerable advantage if the denture were left a little low. He agreed with Mr. Badcock as to the superiority of plaster models. There was a little point about the tray for taking impressions; if the tray was a little deep one got the frænum of the tongue thrust down, giving a false impression. With regard to the turning of the dentures in opposite directions, as referred to by Mr. Brunton, he (Mr. Hern) thought that it was due to the thrust of the swivel being inaccurate, so long as the denture fitted well the plates were correct and retained their adaptation, but when the mouth began to change and the fitting was not quite so correct, then the upper and lower dentures turned round as the consequence of the thrust of the swivel being wrong. It seemed to him that the excellent device brought forward by Mr. Coxon would correct this.

Mr. Coxon had omitted to mention that if one got a slight soreness of the mouth it could be relieved by shifting one of the bolts a little. His contrivance also afforded an opportunity to the patient of seeing if he could do without springs.

Mr. Betts stated that he found it very useful to ask a patient to protrude the tongue; by this means, in conjunction with a shallow tray, the floor of the mouth was raised and one got a much more satisfactory impression.

Mr. Storer Bennett, after the exceedingly interesting and able manner in which Mr. Hepburn had introduced the subject, wished only to touch upon an anatomical point, to which allusion had been made in the opening. In going through the museum certain variations in the height to which the *eminentia articularis* was raised would be observed. He gathered that Mr. Hepburn was of opinion that a good deal of the forward movement of the lower jaw in old people was due to the fact that the *eminentia articularis* in them was much lower than in people of middle life. This view should not go forth on the authority of the Society without a reservation. He (Mr. Bennett) thought that the protrusion was probably due more to the fact of the ligaments yielding and the jaw becoming exceedingly lax in old age than to any absorption and flattening of the *eminentia articularis* itself—*London Dental Record.*

